# The Reciprocal Relationships Among Impulsivity, Self-Control, Gaming Time, and Internet Gaming Disorder Among Adolescents: Prospective Longitudinal Cohort Study

**DOI:** 10.2196/86982

**Published:** 2026-09-23

**Authors:** Yanqiu Yu, Lijing Liu, Jianxin Zhang, Ji-bin Li, Joseph TF Lau

**Affiliations:** 1 School of Public Health, Fudan University, Shanghai, China; 2 West School of Public Health, Sichuan University, Chengdu, China; 3 Department of Clinical Research, Sun Yat-sen University Cancer Center, Guangzhou, China; 4 State Key Laboratory of Oncology in South China, Sun Yat-sen University Cancer Center, Guangzhou, China; 5 Collaborative Innovation Center for Cancer Medicine, Sun Yat-sen University Cancer Center, Guangzhou, China; 6 Zhejiang Provincial Clinical Research Center for Mental Disorders, The Affiliated Wenzhou Kangning Hospital, Wenzhou Medical University, 1 Shengjin Rd, Huanglong Residential District, Wenzhou, China, +86 0577 8182 9200; 7 Public Mental Health Center, School of Mental Health, Wenzhou Medical University, Wenzhou, Zhejiang, China

**Keywords:** internet gaming disorder, impulsivity, self-control, gaming time, cross-lagged panel model, adolescence

## Abstract

**Background:**

Gaming time, impulsivity, and self-control are known determinants of internet gaming disorder (IGD). However, their reciprocity has not been empirically tested.

**Objective:**

This study investigated the reciprocal relationships among gaming time, impulsivity, self-control, and IGD simultaneously within a single cross-lagged panel model among Chinese adolescents, hypothesizing that all 4 variables would be prospectively associated with each other.

**Methods:**

This 1-year, 2-wave prospective longitudinal cohort (observational) study recruited grade 7 and grade 8 students from a convenience sample of 6 junior high schools in Chengdu and Guangzhou, China. Students completed an identical self-administered questionnaire in classrooms (using paper and pencil, without teachers present) in December 2018 (wave 1 [W1]) and December 2019 (wave 2 [W2]). Impulsivity, self-control, gaming time, and IGD were assessed using validated scales. The 2238 students (mean age 12.60, SD 0.63 years, 95% CI 12.58‐12.63; male: n=1102, 49.24%, 95% CI 47.27%‐51.21%) who completed the W1 survey were included in the final data analysis, with multiple imputations addressing missing data from dropouts and unmatched assessments across the 2 study waves.

**Results:**

Adjusting for background characteristics, autoregressive correlations, and concurrent correlations, the cross-lagged panel model showed that (1) the prospective association between gaming time at W1 and IGD at W2 was statistically nonsignificant (β=0.02, 95% CI −0.04 to 0.08), but IGD at W1 was prospectively and positively associated with gaming time at W2 (β=0.08, 95% CI 0.01‐0.16; *P*=.02); (2) self-control at W1 was prospectively and negatively associated with both gaming time at W2 (β=−0.13, 95% CI −0.19 to −0.07; *P*<.001) and IGD at W2 (β=−0.11, 95% CI −0.17 to −0.05; *P*<.001), and IGD at W1 was prospectively and negatively associated with self-control at W2 (β=−0.09, 95% CI −0.15 to −0.03; *P*<.001), but gaming time at W1 was not associated with self-control at W2 (β=0.04, 95% CI −0.01 to 0.09); and (3) the prospective associations between impulsivity at W1 and IGD at W2 (β=0.03, 95% CI −0.03 to 0.09) and between impulsivity at W1 and gaming time at W2 (β=−0.04, 95% CI −0.10 to 0.03) were statistically nonsignificant; reciprocally, neither IGD at W1 (β=0.05, 95% CI −0.01 to 0.11) nor gaming time at W1 (β=−0.01, 95% CI −0.07 to 0.05) was prospectively associated with impulsivity at W2.

**Conclusions:**

This prospective longitudinal cohort study is the first to disentangle the reciprocal prospective associations among gaming time, impulsivity, self-control, and IGD simultaneously within a single cross-lagged panel model. Unlike prior studies, which examined these variables separately and largely cross-sectionally, this design clarified their bidirectionality and relative longitudinal importance. The findings revealed novel differential associations among the 4 variables, findings that promise to reorient the field from gaming time toward the cognitive and motivational processes regulating gaming behaviors. Practically, if replicated in studies with more robust designs, prevention and control programs for IGD should prioritize strengthening adolescents’ self-control rather than merely restricting their gaming time.

## Introduction

### Problem and Review of Relevant Scholarship

Internet gaming disorder (IGD) was considered a condition warranting further research and clinical attention in the *DSM-5 (Diagnostic and Statistical Manual of Mental Disorders, Fifth Edition*) in 2013 [1]. Gaming disorder in terms of both online and offline video gaming was included officially as a mental health condition in the *International Classification of Diseases, 11th Revision* (*ICD-11*) in 2019 [2], which indicates that IGD has become a global health concern. Adolescents are considered a high-risk population for IGD. The prevalence of IGD ranges from 7.5% to 10.0% among adolescents globally; it is higher in China, ranging from 8.7% to 12.9% [3]. A wide variety of adverse consequences resulting from adolescent IGD have been reported in terms of psychological (eg, depression) [4,5], psychosocial (eg, social anxiety and worsened parent-child relationships) [6,7], physical (eg, insomnia and impaired neurocognitive functions) [8-11], and behavioral (eg, delinquency) [12] domains.

Gaming time is considered an indicator of IGD in recent publications [13], possibly due to its strong associations with IGD [14]. However, intensive gaming does not equate to IGD, with emerging evidence that intensive gamers do not develop IGD [15-19] or experience gaming-related negative consequences [20-23]. Furthermore, the associations between gaming time and IGD are mixed, with some empirical studies reporting significant positive associations [24-27] and others reporting nonsignificant associations [17,28,29]. Hence, the effect of gaming time on IGD needs confirmation. On the other hand, tolerance in IGD refers to the psychological need to increase gaming time [30]; it is considered a symptom of IGD in the *DSM-5* [1] and implies that those with IGD would increase their gaming time. Thus, IGD might be reversely and positively associated with gaming time. Case-control studies support this postulation that people with IGD tend to spend more time gaming than recreational gamers [31]. These findings suggest a reciprocal relationship between gaming time and IGD. To the best of our knowledge, only 1 longitudinal study, which was conducted among Dutch adolescents, has reported that gaming frequency did not predict IGD, but that IGD predicted gaming frequency [32].

Impulsivity and self-control are 2 other important determinants of addictive behaviors among adolescents, including substance use [33-35], alcohol misuse [36], smoking conventional and electronic cigarettes [37,38], and IGD [39]. According to the dual-process theories, impulsivity and self-control represent 2 distinct processes affecting decision-making related to risky behaviors [40-47]. Impulsivity is characterized by a reactive, intuitive, and affective process that exhibits a strong sensitivity to temptations, leading to immediate actions without thoughtful consideration [46]. In contrast, self-control involves a reasoned process and the ability to deliberately regulate impulses [48-51]. Accordingly, individuals with stronger impulsivity tend to respond to gaming cues with heightened spontaneity, while those with weaker self-control may find it more difficult to resist gaming temptations and stop gaming. Both scenarios could plausibly lead to increased gaming time and heightened risk of IGD, as supported by empirical evidence [52-55]. In addition, the dual-process theory supports the reverse associations between IGD and impulsivity or self-control. IGD is characterized by compulsive engagement, loss of control, and continued use despite the presence of adverse consequences [1]. Theoretically, sustained engagement in addictive behaviors could progressively undermine regulatory resources through repetitive failure experience, habituation of inhibitory processes, and neurological changes due to continued reward seeking [44-47]. Therefore, IGD cases may weaken self-control due to depleted regulatory mechanisms that moderate impulsive responses. Case-control studies empirically support this postulation, reporting that individuals with IGD showed stronger impulsivity and weaker self-control than those without IGD [54,56]. These findings suggest the bidirectional relationships between impulsivity or self-control and IGD. To our knowledge, only 1 longitudinal study has investigated the bidirectional relationship between self-control and IGD among Chinese children and adolescents, finding that self-control was neither the cause nor the outcome of IGD [57].

### Hypothesis, Aims, and Objectives

The existing evidence has several important gaps. First, the reciprocal relationships between gaming time, impulsivity, self-control, and IGD have rarely been investigated using a prospective longitudinal cohort study design. Second, the few available longitudinal studies examined these associations in isolation (eg, the association between self-control and IGD alone), rather than simultaneously in a single model that can account for their shared variance. Third, only 1 relevant longitudinal study has been conducted among Chinese adolescents, reflecting the limited empirical evidence. Hence, this study aimed to fill these knowledge gaps by simultaneously examining the prospective reciprocal relationships among impulsivity, self-control, gaming time, and IGD among Chinese adolescents in a 12-month prospective longitudinal cohort study. In total, five research hypotheses were tested regarding reciprocal relationships: (1) between gaming time and IGD, (2) between impulsivity and gaming time, (3) between impulsivity and IGD, (4) between self-control and gaming time, and (5) between self-control and IGD.

## Methods

### Conditions and Design

This study used a prospective longitudinal cohort design with 2 assessment waves (a 2-wave panel design) and a 12-month measurement interval. This study was a nonexperimental, observational study; no conditions were experimentally manipulated, and all variables were naturally observed.

### Inclusion and Exclusion

The target population comprised junior middle school students. Inclusion criteria were (1) enrolled in grade 7 or 8, (2) being aged 12 to 14 years, and (3) willing to participate and provide informed consent. Grade 9 students were excluded, as they would have taken the public entrance examination for senior middle school and left school before the end of the 12-month follow-up period.

### Sampling Procedure

A 12-month prospective cohort study was conducted in Guangzhou and Chengdu, China; the baseline and follow-up surveys were conducted in December 2018 (wave 1 [W1]) and December 2019 (wave 2 [W2]), respectively. Four junior middle schools from Guangzhou and 2 from Chengdu were selected by convenience sampling, and all grade 7 and grade 8 students at the participating schools were invited to participate in this study.

### Ethical Considerations

This study was approved by the Survey and Behavioral Research Ethics Committee of the Chinese University of Hong Kong (SBRE-18-430). Participation in this study was entirely voluntary. Before data collection, well-trained field workers briefed the students on the objective, content, and logistics of the study. The students were also informed that participation was voluntary and that they had the right to refuse to participate at any time without any negative consequences. Written informed consent was not obtained to maintain anonymity; instead, informed consent was implied by the voluntary completion and submission of the questionnaire. The above briefing information was also printed on the cover page of the questionnaire.

### Data Collection

All collected data were anonymized, and no direct personal identifiers are included in this manuscript or its supplementary materials. No financial or material incentives were provided to participants for their involvement in this study. In addition, parental approval was sought using a parental opt-out procedure.

### Sample Size, Power, and Precision

Sample size planning was conducted based on the cross-lagged effects among impulsivity, self-control, gaming time, and IGD across the 2 waves. Assuming the conventional small standardized cross-lagged effect of 0.10, autoregressive stabilities of 0.50, and intercorrelations of 0.30 among the 4 variables, the Monte Carlo simulation (4000 replications) estimated an intended sample size of 640 participants, with the power of 0.80, 2-tailed Cronbach α of 0.05, and expected 95% CI of ±0.07. After further taking into account the attrition rate of about 20%, the baseline sample size was estimated to be approximately 800 participants. The final sample size in this study was 2238 participants, which was sufficient.

### Measures and Covariates

#### IGD

The 9-item *DSM-5* checklist was used to assess IGD [1]; it recorded the presence of addictive symptoms, including preoccupation, withdrawal, tolerance, inability to control gaming, loss of interest in other activities, continued gaming despite psychological and/or social problems, deception, avoidance, and significant loss due to internet gaming. IGD is defined by the endorsement of 5 or more items in the past 12 months (yes or no response options) [58]. The Chinese version of the *DSM-5* has been validated in Chinese adolescents with satisfactory psychometric properties [59]. The Cronbach α of the checklist was 0.77 at both the W1 and W2 surveys in this study. The summative score of the checklist was treated as a continuous variable in this study.

#### Impulsivity

Impulsivity was assessed using the 10-item motor subscale of the Barratt Impulsiveness Scale, which indicates a tendency to act on the spur of the moment and have fast reactions [60]. The Chinese version makes some cultural adaptations and shows good reliability and construct validity in Chinese adolescents [61]. A sample item is “I do things without thinking.” The items were rated on a 5-point Likert scale (1=completely disagree to 5=completely agree); higher scores indicate higher levels of impulsivity (a continuous variable). The Cronbach α of the scale was 0.89 at W1 and 0.92 at W2 in the present study.

#### Self-Control

Self-control was assessed using the 13-item Brief Self-Control Scale [50], which demonstrates good psychometric properties in Chinese adolescents [62]. A sample item is “I am good at resisting temptation.” The items were rated on a 5-point Likert scale (1=never to 5=always); higher scores indicated higher levels of self-control (a continuous variable). The Cronbach α of the scale was 0.76 at W1 and 0.74 at W2 in the present study.

#### Gaming Time

The average gaming time in the past month (response options: <2 hours; between 2‐6, 6‐10, 10‐20, 20‐30, 30‐40, and 40‐50 hours; and >50 hours a week) [63,64] was assessed. This categorical variable was transformed into a continuous variable based on the midpoints of the respective time intervals of the response categories, with the last category (>50 hours) coded as 55 hours [65,66].

#### Background Factors

Background information was collected, including sex, age, study site, whether born in the study site, whether living with both parents, both father’s and mother’s educational levels (junior middle school or below, senior middle school or equal, or college or above), and self-reported family financial situation (below average, average, or above average).

### Analytic Strategy

The Little missing completely at random (MCAR) test was conducted to evaluate the pattern of missing data due to dropouts; multiple imputation (with 20 imputed datasets) was used to address missing data from dropouts and matched assessments at both W1 and W2. Normality tests (using the Kolmogorov-Smirnov and Shapiro-Wilk tests), within-individual comparisons by survey time, and Spearman correlation analysis (with Benjamini-Hochberg false discovery rate correction) were examined for the key variables. Data analyses were performed in SPSS (version 26.0; IBM Corp). The hypothesized cross-lagged panel model was conducted in Mplus (version 8.0; Muthén and Muthén). The cross-lagged panel analysis was selected because it is specifically designed to examine the prospective effects of one construct on another (eg, the effect of self-control at W1 on IGD at W2) while controlling for autoregressive effects (ie, the stability of the same construct across time points, such as the correlation between IGD at W1 and IGD at W2) and the concurrent correlations among variables at the same time point [67]. This makes it a well-suited and widely used method for testing reciprocal relationships, as it explicitly adjusts for the autoregressive variance that simpler approaches (eg, standard regression or linear mixed models) do not account for directly [67]. The robust maximum likelihood estimator was used in the panel model; satisfactory model fit indices included comparative fit index (CFI) ≥0.90, root mean square error of approximation (RMSEA) ≤0.08, and standardized root mean square residual (SRMR) ≤0.08 [68]. Statistical significance was defined as 2-tailed *P*<.05 or 95% CI of the unstandardized or standardized coefficients not involving 0.

## Results

### Participant Characteristics

Baseline participant characteristics are presented in Table 1. The mean age of the adolescents was 12.60 (SD 0.63, 95% CI 12.58-12.63 years); 50.67% (1136/2238; 95% CI 48.79%-52.73%) were female, and most were born in the study city (1829/2238, 81.72%; 95% CI 79.98%-83.35%) and lived with both parents (1829/2238, 81.72%; 95% CI 79.98%-83.35%); 26.01% (582/2238; 95% CI 24.18%-27.92%) self-reported a poor or very poor family financial situation.

**Table 1.** Baseline characteristics of 2238 adolescents participating in a 2-wave prospective longitudinal cohort study examining the reciprocal relationships among impulsivity, self-control, gaming time, and internet gaming disorder in 2 Chinese cities, December 2018 to December 2019 (N=2238).

	Adolescents, n (%; 95% CI)
Study city	
Guangzhou	1170 (50.04; 48.07‐52.01)
Chengdu	1168 (49.96; 47.99‐51.93)
School	
School A	256 (11.44; 10.16‐12.86)
School B	439 (19.62; 17.97‐21.37)
School C	183 (8.18; 7.08‐9.43)
School D	292 (13.05; 11.69‐14.54)
School E	604 (26.99; 25.13‐28.93)
School F	464 (20.73; 19.03‐22.54)
Sex	
Female	1136 (50.76; 48.79‐52.73)
Male	1102 (49.24; 47.27‐51.21)
Born in the study city	
No	409 (18.28; 16.65‐20.02)
Yes	1829 (81.72; 79.98‐83.35)
Living with both parents	
Yes	1829 (81.72; 79.98‐83.35)
No	409 (18.28; 16.65‐20.02)
Father’s educational level	
Secondary school or below	992 (44.33; 42.36‐46.31)
Senior middle school	653 (29.18; 27.30‐31.12)
College or above	593 (26.50; 24.66‐28.41)
Mother’s educational level	
Secondary school or below	1006 (44.95; 42.98‐46.93)
Senior middle school	657 (29.36; 27.47‐31.30)
College or above	575 (25.69; 23.87‐27.59)
Family financial situation	
Poor or very poor	582 (26.01; 24.18‐27.92)
Moderate	1418 (63.36; 61.42‐65.26)
Good or very good	238 (10.63; 9.40‐12.01)

### Participant Flow

**Figure 1.**
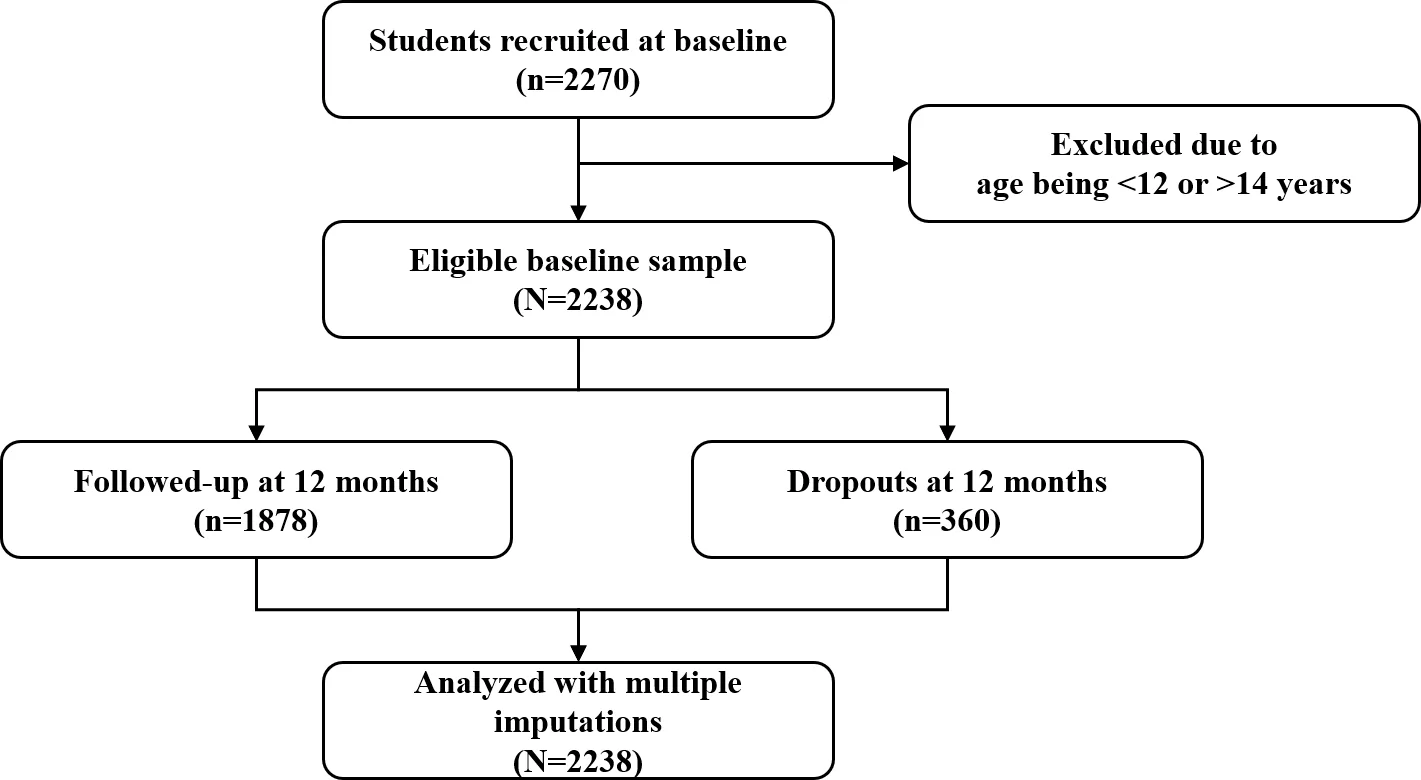
Flowchart of participant recruitment, follow-up, and analysis in a 2-wave prospective longitudinal cohort study examining the reciprocal relationships among impulsivity, self-control, gaming time, and internet gaming disorder among adolescents in 2 Chinese cities, December 2018 to December 2019.

Of the 2270 students who completed the baseline questionnaire, 32 (1.4%) were removed due to falling out of the age range of 12 to 14 years. The baseline and follow-up questionnaires completed by each student were matched based on a unique personal code (ie, the birthday; the last 4 digits of his or her father’s and mother’s mobile phone numbers, respectively; and the last 2 letters of his or her father’s and mother’s names, respectively); a total of 1878 completed follow-up questionnaires were matched (Figure 1). Compared with students who completed the follow-up assessment, those lost to follow-up were more likely to be from Chengdu, female, not born in the study site, not living with both parents, have fathers and mothers with an educational level of junior middle school or below, and report a poor or very poor family financial situation. They were also older and exhibited more IGD symptoms, higher impulsivity, lower self-control, and longer gaming time (Multimedia Appendix 1). The Little MCAR test was statistically significant (*χ*^2^_352_=882.7; *P*<.001), suggesting that the data were not missing completely at random. Multiple imputation (m=20) was subsequently conducted to address missing data.

### Within-Individual Comparisons in Key Variables Across 2 Waves

Normality tests demonstrated that all key variables (impulsivity, self-control, gaming time, and IGD) at W1 and W2 violated normality assumptions (all *P* values <.001). Compared with W1, participants at W2 exhibited higher impulsivity (*P*<.001; Cohen *d*=0.10), weaker self-control (*P*<.001; Cohen *d*=0.24), longer gaming time (*P*<.001; Cohen *d*=0.04), and more severe IGD symptoms (*P*<.001; Cohen *d*=0.07; Table 2).

**Table 2.** Within-participant changes in key variables between wave 1 (December 2018) and wave 2 (December 2019) in a 2-wave prospective longitudinal cohort study examining the reciprocal relationships among impulsivity, self-control, gaming time, and internet gaming disorder (IGD) among adolescents in 2 Chinese cities.

	Range	Wave 1^a^, mean (SD; 95% CI)	Wave 2^b^, mean (SD; 95% CI)	*P* value	Cohen *d*
Impulsivity score	11‐55	22.02 (7.23; 21.73‐22.31)	22.70 (7.73; 22.39‐23.01)	<.001	0.10
Self-control score	13‐65	43.01 (7.81; 42.70‐43.32)	41.91 (7.41; 41.61‐42.21)	<.001	0.24
Gaming time (hours)	0‐55	3.36 (8.08; 3.03‐3.69)	5.03 (10.00; 4.62‐5.44)	<.001	0.04
IGD score	0‐9	1.96 (2.19; 1.87‐2.05)	2.11 (2.24; 2.02‐2.20)	<.001	0.07

a Assessed at baseline.

b Assessed at follow-up.

### Spearman Correlation Analysis

Spearman correlation analysis showed that impulsivity, self-control, gaming time, and IGD were significantly correlated with each other at the same time points (ie, W1 or W2) and across time points (all adjusted *P* values <.01). Specifically, impulsivity at W1 and W2 was positively correlated with gaming time at both waves (ρ ranged from 0.08 to 0.20) and IGD at both waves (ρ ranged from 0.23 to 0.45), while self-control at both W1 and W2 was negatively correlated with gaming time (ρ ranged from −0.23 to −0.12) and IGD (ρ ranged from −0.44 to −0.27) at both waves. Gaming time at both waves was also positively correlated with IGD at both waves (ρ ranged from 0.18 to 0.28; Table 3).

**Table 3.** Spearman correlations among impulsivity, self-control, gaming time, and internet gaming disorder (IGD) in a 2-wave prospective longitudinal cohort study of 2238 adolescents from 2 Chinese cities, December 2018 to December 2019.

Variables	Impulsivity (W1)	Self-control (W1)	Gaming time (W1)	IGD (W1)	Impulsivity (W2)	Self-control (W2)	Gaming time (W2)
Impulsivity (W1^a^), ρ (95% CI)	—^b^	—	—	—	—	—	—
Self-control (W1), ρ (95% CI)	−0.55 (−0.58 to −0.52)	—	—	—	—	—	—
Gaming time (W1), ρ (95% CI)	0.20 (0.16 to 0.24)	−0.23 (−0.27 to −0.19)	—	—	—	—	—
IGD (W1), ρ (95% CI)	0.39 (0.36 to 0.42)	−0.44 (−0.47 to −0.41)	0.28 (0.24 to 0.32)	—	—	—	—
Impulsivity (W2^c^), ρ (95% CI)	0.42 (0.39 to 0.45)	−0.32 (−0.36 to −0.28)	0.09 (0.05 to 0.13)	0.23 (0.19 to 0.27)	—	—	—
Self-control (W2), ρ (95% CI)	−0.33 (−0.37 to −0.29)	0.43 (0.40 to 0.46)	−0.12 (−0.16 to −0.08)	−0.27 (−0.31 to −0.23)	−0.55 (−0.58 to −0.52)	—	—
Gaming time (W2), ρ (95% CI)	0.08 (0.04 to 0.12)	−0.19 (−0.23 to −0.15)	0.25 (0.21 to 0.29)	0.18 (0.14 to 0.22)	0.08 (0.04 to 0.12)	−0.13 (−0.17 to −0.09)	—
IGD (W2), ρ (95% CI)	0.26 (0.22 to 0.30)	−0.30 (−0.34 to −0.26)	0.20 (0.16 to 0.24)	0.45 (0.42 to 0.48)	0.35 (0.31 to 0.39)	−0.39 (−0.42 to −0.36)	0.25 (0.21 to 0.29)

a Assessed at baseline.

b Not applicable.

c Assessed at follow-up.

### Cross-Lagged Panel Analysis

**Figure 2.**
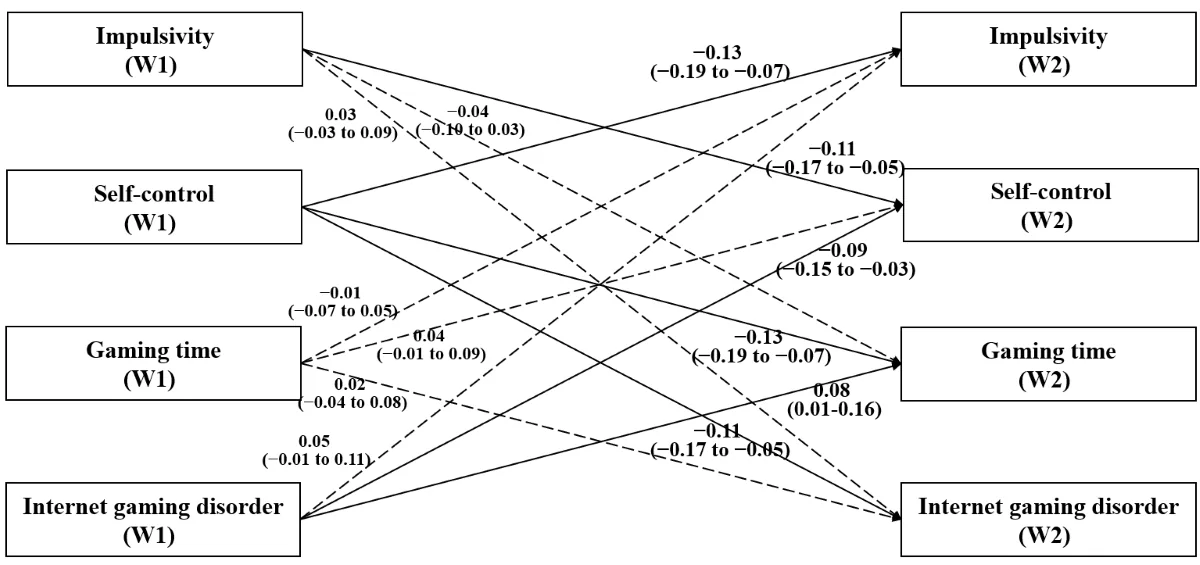
Cross-lagged panel model examining the reciprocal relationships among impulsivity, self-control, gaming time, and internet gaming disorder in a 2-wave prospective longitudinal cohort study of 2238 adolescents from 2 Chinese cities, December 2018 to December 2019. Standardized path coefficients are shown as β and 95% CIs. Solid lines indicate statistically significant paths, and dashed lines indicate nonsignificant paths. Wave 1 (W1) represents the baseline assessment, and wave 2 (W2) represents the 12-month follow-up assessment. The model was adjusted for background factors, including school, age, sex, whether the adolescent was born in the study site, whether they were living with both parents, the father’s educational level, the mother’s educational level, and the family financial situation*.*

Figure 2 and Multimedia Appendix 2 present the hypothesized cross-lagged panel model with adjustment for background factors, autoregression correlations, and concurrent correlations; the model showed satisfactory model fit indices: CFI=0.91, RMSEA=0.04, and SRMR=0.04. Only self-control at W1 was significantly and prospectively associated with IGD at W2 (β=−0.11, 95% CI −0.17 to −0.05); neither impulsivity at W1 (β=0.03, 95% CI −0.03 to 0.09) nor gaming time at W1 (β=0.02, 95% CI −0.04 to 0.08) were associated with IGD at W2. In contrast, IGD at W1 was significantly and prospectively associated with self-control at W2 (β=−0.09, 95% CI −0.15 to −0.03) and gaming time at W2 (β=0.08, 95% CI 0.01-0.16), but not impulsivity at W2 (β=0.05, 95% CI −0.01 to 0.11). In addition, self-control at W1 (β=−0.13, 95% CI −0.19 to −0.07), but not impulsivity at W1 (β=−0.04, 95% CI −0.10 to 0.03), was prospectively associated with gaming time at W2, although gaming time at W1 was not significantly associated with either impulsivity (β=−0.01, 95% CI −0.07 to 0.05) or self-control (β=0.04, 95% CI −0.01 to 0.09) at W2. Moreover, impulsivity at W1 was prospectively associated with self-control at W2 (β=−0.11, 95% CI −0.17 to −0.05), and self-control at W1 was also prospectively associated with impulsivity at W2 (β=−0.13, 95% CI −0.19 to −0.07).

## Discussion

### Principal Findings

This 2-wave prospective longitudinal cohort study extends prior research by simultaneously examining the reciprocal relationships among 4 theoretically relevant variables—impulsivity, self-control, gaming time, and IGD—in a single cross-lagged panel model. This allows for direct comparisons regarding the relative prospective associations of impulsivity and self-control with IGD and gaming time, when they were controlled for one another. Key findings indicated that (1) IGD at W1 was prospectively associated with gaming time at W2, but the reverse association was statistically nonsignificant; (2) self-control, but not impulsivity, at W1 was prospectively associated with both gaming time and IGD at W2; (3) IGD at W1 was prospectively associated with self-control, but not impulsivity, at W2; and (4) gaming time at W1 was not significantly associated with either impulsivity or self-control at W2.

This study explored the reciprocal relationships between gaming time and IGD among Chinese adolescents. The results found that IGD at W1 was prospectively and positively associated with gaming time at W2, but not vice versa, which partially supports our hypotheses and corroborates a previous study conducted among Dutch adolescents [32]. The nonsignificant prospective association between gaming time at W1 and IGD at W2, along with the nonsignificant associations between gaming time at W1 and impulsivity and self-control at W2, aligns with emerging evidence that intensive gaming does not necessarily increase IGD severity or associated negative consequences [17-19,22,23]. Moreover, the impacts of gaming time on IGD were found to weaken over a 2-year follow-up period among Singaporean children and adolescents [69]. While most empirical evidence has reported positive associations between gaming time and IGD [26,27], the inconsistencies between the findings of this and previous studies may be due to the existence of potential moderators affecting the dosage effect of gaming time on IGD. For instance, the association between gaming time and IGD was stronger among adolescents with stronger perceived urges and loneliness [14]. However, it was beyond the scope of this study to examine these potential moderation effects, highlighting the need for future studies. Nonetheless, the observed reciprocal associations between gaming time and IGD in this study suggest that elevated gaming time may be more plausibly understood as a correlate or consequence of IGD rather than a driver of its onset or maintenance. Future studies should examine whether interventions targeting gaming time could reduce IGD symptoms and identify the subgroups in which such interventions are most effective.

This study also revealed the differential reciprocal associations between impulsivity and self-control and IGD. The bidirectional relationships between impulsivity and IGD were statistically nonsignificant. This suggests that, although stronger impulsivity was associated with excessive gaming [44-47], impulsivity did not have incremental prospective associations with IGD when self-control, gaming time, and IGD at W1 were simultaneously controlled for. However, notably, the cross-lagged panel model estimated the total (cross-lagged) effects between variables and did not decompose them into direct and indirect effects [70]; questions about whether impulsivity operates through mediators (eg, maladaptive cognitions [39]) remain open and require further examination with appropriate longitudinal designs. In contrast, the bidirectional associations between self-control and IGD were statistically significant. Specifically, lower self-control at W1 was prospectively associated with more IGD symptoms at W2, while more IGD symptoms at W1 were prospectively associated with lower self-control at W2. Although both associations were statistically significant, it is important to note that the latter effect was small in magnitude, and its clinical and practical significance remains uncertain. Accordingly, while these associations are consistent with the theoretical proposition that self-control and IGD may reinforce one another across development [71], the small effect size of the association between IGD at W1 and self-control at W2 warrants cautious interpretation. Whether these associations reflect true reinforcing processes requires further investigation in future longitudinal studies with more robust designs (eg, more than 2 assessment waves or quasi-experiments).

This study also found that higher self-control at W1 was prospectively associated with lower impulsivity and shorter gaming time at W2. These associations are consistent with the theoretical role of self-control as a regulatory resource [72], although they do not confirm that strengthening self-control would causally reduce impulsivity or gaming time. Nonetheless, the observed prospective associations suggest that self-control may be a relevant target for future IGD interventions. If replicated in experimental studies, established approaches to strengthening self-control may merit investigation, including cognitive behavioral therapy, which emphasizes the development of coping strategies, problem-solving skills, and impulse control skills [73], as well as goal setting and planning in a progressive, manageable manner [74]. Mindfulness-based training has also shown promising effects on improving adolescents’ self-awareness and self-regulation skills [75,76] and may similarly need evaluation in future IGD intervention studies.

The present study has several limitations. First, there was a relatively high attrition rate in this prospective longitudinal cohort study, and some background characteristics between participants who completed the follow-up assessment and those who were lost to follow-up were statistically significant (Multimedia Appendix 1), although multiple imputations were used to address missing data from dropouts and W1 or W2 assessments, and the cross-lagged panel model was adjusted for background variables. Second, there might have been selection bias, as the participating schools were selected based on convenience from 2 Chinese cities. Generalization of the results of this study to other regions or countries should hence be done cautiously. Third, as the questionnaire was self-administered, reporting bias might have occurred. For instance, recall bias might have occurred when students self-reported their gaming time. However, using categorical response options and the midpoint translation might have reduced such bias to some extent [77]. Fourth, the cross-lagged panel model included participants with varying IGD statuses at W1. The cross-lagged effects therefore reflect average prospective associations across all transitions from W1 to W2 and should not be interpreted as directly addressing questions related to the onset or maintenance of IGD. Finally, this study comprised only 2 waves of assessments, which limits the strength of longitudinal inference relative to studies with 3 or more waves. Future longitudinal studies with more waves and shorter time lags are needed to verify the findings and consider within-person changes.

### Conclusions

In conclusion, this study advances understanding of the prospective associations among gaming time, impulsivity, self-control, and IGD among Chinese adolescents by simultaneously examining all 4 constructs in a single 2-wave cross-lagged panel model. The results reveal differential patterns. First, IGD at W1 was prospectively associated with greater gaming time at W2, but not vice versa. If replicated, this is a potentially important reorientation for future studies on IGD development, which may look beyond gaming time toward the motivational and cognitive processes regulating the gaming experience and behaviors. Second, self-control showed significant bidirectional prospective associations with IGD, whereas impulsivity did not. This divergence between impulsivity and self-control in their prospective associations with IGD challenges the assumptions of common dual-process theories that both constructs carry equivalent longitudinal relevance [78]. It also calls for greater caution in future IGD research, as correlated constructs in cross-sectional studies may perform differently when examined in prospective studies or in combination.

## Supplementary material

10.2196/86982 Multimedia Appendix 1 Attrition analysis of participants in a 2-wave prospective longitudinal cohort study examining the reciprocal relationships among impulsivity, self-control, gaming time, and internet gaming disorder among 2238 adolescents from 2 Chinese cities, December 2018 to December 2019.

10.2196/86982 Multimedia Appendix 2 Cross-lagged panel analysis examining the reciprocal relationships among impulsivity, self-control, gaming time, and internet gaming disorder in a 2-wave prospective longitudinal cohort study of 2238 adolescents from 2 Chinese cities, December 2018 to December 2019.

## Abbreviations:

CFI: comparative fit index
*DSM-5*: *Diagnostic and Statistical Manual of Mental Disorders, Fifth Edition*
*ICD-11*: *International Classification of Diseases, 11th revision*
IGD: internet gaming disorder
MCAR: missing completely at random
RMSEA: root mean square error of approximation
SRMR: standardized root mean square residual
