# Supplementary material for: The Reciprocal Relationships Among Impulsivity, Self-Control, Gaming Time, and Internet Gaming Disorder Among Adolescents: Prospective Longitudinal Cohort Study
Source: JMIR Serious Games. 2026 Sep 23;14:e86982. doi: 10.2196/86982 (PMC13600582; doi:10.2196/86982)
Supplement: Supplementary file 1 [file games-v14-e86982-s001.docx]

Table S1 Attrition analysis of the 2-wave longitudinal study on the reciprocal relationships among impulsivity, self-control, gaming time, and internet gaming disorder (IGD) among 2238 adolescents from two Chinese cities from December 2018 to December 2019

|  | Followed up  (n=1878) | | Lost to follow up  (n=360) | | *P^#^* |
| --- | --- | --- | --- | --- | --- |
|  | n | % (95% CI) | n | % (95% CI) |  |
| **Background factors** |  |  |  |  |  |
| Study site |  |  |  |  | <.001 |
| Guangzhou | 1170 | 62.3 (60.1-64.4) | 0 | 0 (0-1) |  |
| Chengdu | 708 | 37.7 (35.6-39.9) | 360 | 100 (99-100) |  |
| School |  |  |  |  | <.001 |
| School A | 256 | 13.6 (12.1-15.3) | 0 | 0 (0-1) |  |
| School B | 439 | 23.4 (21.5-25.4) | 0 | 0 (0-1) |  |
| School C | 183 | 9.7 (8.4-11.2) | 0 | 0 (0-1) |  |
| School D | 292 | 15.5 (13.9-17.3) | 0 | 0 (0-1) |  |
| School E | 396 | 21.1 (19.3-23) | 208 | 57.8 (52.5-62.9) |  |
| School F | 312 | 16.6 (15-18.4) | 152 | 42.2 (37.1-47.5) |  |
| Age (years) | Mean 12.6 (95% CI: 12.5-12.6),  SD 0.6 | | Mean 12.8 (95% CI: 12.7-12.9), SD 0.7 | | <.001 |
| Sex |  |  |  |  | .049 |
| Female | 916 | 48.8 (46.5-51) | 201 | 55.8 (50.5-61) |  |
| Male | 928 | 49.4 (47.2-51.6) | 153 | 42.5 (37.4-47.8) |  |
| Missing data | 34 | 1.8 (1.3-2.6) | 6 | 1.7 (0.7-3.7) |  |
| Whether born in the study site |  |  |  |  | <.001 |
| No | 310 | 16.5 (14.9-18.3) | 89 | 24.7 (20.3-29.7) |  |
| Yes | 1528 | 81.4 (79.6-83.0) | 255 | 70.8 (65.8-75.4) |  |
| Missing data | 40 | 2.1 (1.5-2.9) | 16 | 4.4 (2.6-7.2) |  |
| Living arrangement |  |  |  |  | <.001 |
| Others | 296 | 15.8 (14.1-17.6) | 99 | 27.5 (22.9-32.6) |  |
| With both parents | 1525 | 81.2 (79.4-82.9) | 249 | 69.2 (64.1-73.9) |  |
| Missing data | 57 | 3.0 (2.2-4.1) | 12 | 3.3 (1.8-5.8) |  |
| Father’s educational level |  |  |  |  | <.001 |
| Junior middle school or below | 742 | 39.5 (37.3-41.8) | 186 | 51.7 (46.4-56.9) |  |
| Senior middle school | 497 | 26.5 (24.5-28.5) | 109 | 30.3 (25.5-35.5) |  |
| College or above | 510 | 27.2 (25.1-29.3) | 32 | 8.9 (6.2-12.5) |  |
| Missing data | 129 | 6.9 (5.7-8.2) | 33 | 9.2 (6.5-12.8) |  |
| Mother’s educational level |  |  |  |  | <.001 |
| Junior middle school or below | 757 | 40.3 (38.1-42.6) | 180 | 50.0 (44.7-55.3) |  |
| Senior middle school | 509 | 27.1 (25.1-29.3) | 96 | 26.7 (22.2-31.7) |  |
| College or above | 484 | 25.8 (23.8-27.9) | 43 | 11.9 (8.8-16.0) |  |
| Missing data | 128 | 6.8 (5.6-8.2) | 41 | 11.4 (8.4-15.3) |  |
| Household financial situation |  |  |  |  | .04 |
| Good/very good | 498 | 26.5 (24.5-28.6) | 70 | 19.4 (15.4-24.2) |  |
| Moderate | 1150 | 61.2 (59.0-63.4) | 241 | 66.9 (61.8-71.7) |  |
| Poor/very poor | 192 | 10.2 (8.9-11.7) | 42 | 11.7 (8.6-15.7) |  |
| Missing data | 38 | 2.0 (1.4-2.9) | 7 | 1.9 (0.9-4.0) |  |
| **Key variables at baseline** |  |  |  |  |  |
| IGD | Mean 2.0 (95% CI: 2.0-2.2),  SD 2.2 | | Mean 2.9 (95% CI: 2.6-3.3),  SD 2.7 | | .001 |
| Impulsivity | Mean 21.7 (95% CI: 21.4-22),  SD 7.2 | | Mean 23.6 (95% CI: 22.8-24.5), SD 7.9 | | .001 |
| Self-control | Mean 43.2 (95% CI: 42.8-43.5),  SD 7.8 | | Mean 42.2 (95% CI: 41.4-42.9), SD 7.1 | | .03 |
| Gaming time | Mean 3.4 (95% CI: 3.0-3.8),  SD 7.7 | | Mean 5.0 (95% CI: 3.8-6.2),  SD 11.3 | | .002 |

Note. ^#^, Chi-square test and independent-sample t-test were used when applicable.
