# Supplementary material for: The Reciprocal Relationships Among Impulsivity, Self-Control, Gaming Time, and Internet Gaming Disorder Among Adolescents: Prospective Longitudinal Cohort Study
Source: JMIR Serious Games. 2026 Sep 23;14:e86982. doi: 10.2196/86982 (PMC13600582; doi:10.2196/86982)
Supplement: Supplementary file 2 [file games-v14-e86982-s002.docx]

Table S2. Cross-lagged panel analysis testing the reciprocal relationships among impulsivity, self-control, gaming time, and internet gaming disorder (IGD) of the 2-wave longitudinal study among 2238 adolescents from two Chinese cities from December 2018 to December 2019

|  | Unstandardized coefficients  (95% CI) | S.E. | Standardized coefficients  (95% CI) | S.E. |
| --- | --- | --- | --- | --- |
| **Model fit indices** (*df*=70; RMSEA=0.04; CFI=0.91; SRMR=0.04) |  |  |  |  |
| ***Cross-lagged correlations*** |  |  |  |  |
| Self-control (W1) → Impulsivity (W2) | −0.13 (−0.19 to −0.07) | 0.03 | −0.13 (−0.19 to −0.07) | 0.03 |
| Gaming time (W1) → Impulsivity (W2) | −0.01 (−0.06 to 0.05) | 0.03 | −0.01 (−0.07 to0.05) | 0.03 |
| IGD (W1) → Impulsivity (W2) | 0.18 (−0.03 to 0.38) | 0.10 | 0.05 (−0.01 to 0.11) | 0.03 |
| Impulsivity (W1) → Self-control (W2) | −0.11 (−0.17 to −0.05) | 0.03 | −0.11 (−0.17 to −0.05) | 0.03 |
| Gaming time (W1) → Self-control (W2) | 0.03 (−0.01 to 0.08) | 0.02 | 0.04 (−0.01 to 0.09) | 0.03 |
| IGD (W1) → Self-control (W2) | −0.29 (−0.48 to −0.11) | 0.10 | −0.09 (−0.15 to −0.03) | 0.03 |
| Impulsivity (W1) → Gaming time (W2) | −0.05 (−0.13 to 0.03) | 0.04 | −0.04 (−0.10 to 0.03) | 0.03 |
| Self-control (W1) → Gaming time (W2) | −0.16 (−0.23 to −0.09) | 0.04 | −0.13 (−0.19 to −0.07) | 0.03 |
| IGD (W1) → Gaming time (W2) | 0.34 (0.05-0.63) | 0.15 | 0.08 (0.01-0.16) | 0.04 |
| Impulsivity (W1) → IGD (W2) | 0.01 (−0.01 to 0.03) | 0.01 | 0.03 (−0.03 to 0.09) | 0.03 |
| Self-control (W1) → IGD (W2) | −0.03 (−0.05 to −0.01) | 0.01 | −0.11 (−0.17 to −0.05) | 0.03 |
| Gaming time (W1) → IGD (W2) | 0.01 (−0.01 to 0.02) | 0.01 | 0.02 (−0.04 to 0.08) | 0.03 |
|  |  |  |  |  |
| ***Autoregression correlations*** |  |  |  |  |
| Impulsivity (W1) → Impulsivity (W2) | 0.32 (0.26-0.39) | 0.03 | 0.31 (0.25-0.37) | 0.03 |
| Self-control (W1) → Self-control (W2) | 0.33 (0.28-0.38) | 0.03 | 0.35 (0.29-0.40) | 0.03 |
| Gaming time (W1) → Gaming time (W2) | 0.24 (0.17-0.32) | 0.04 | 0.22 (0.15-0.29) | 0.04 |
| IGD (W1) → IGD (W2) | 0.39 (0.32-0.46) | 0.04 | 0.40 (0.33-0.47) | 0.04 |
|  |  |  |  |  |
| ***Concurrent correlations*** |  |  |  |  |
| Impulsivity (W1) − Self-control (W1) | −30.95 (−33.71 to −28.20) | 1.41 | −0.54 (−0.58 to −0.51) | 0.02 |
| Impulsivity (W1) − Gaming time (W1) | 11.66 (8.14-15.17) | 1.79 | 0.19 (0.14-0.24) | 0.03 |
| Impulsivity (W1) − IGD (W1) | 6.44 (5.58-7.30) | 0.44 | 0.38 (0.33-0.42) | 0.02 |
| Self-control (W1) − Gaming time (W1) | −12.04 (−15.26 to −8.81) | 1.64 | −0.18 (−0.23 to −0.14) | 0.02 |
| Self-control (W1) − IGD (W1) | −7.87 (−8.71 to −7.02) | 0.43 | −0.44 (−0.48 to −0.40) | 0.02 |
| Gaming time (W1) − IGD (W1) | 6.56 (5.22-7.89) | 0.68 | 0.33 (0.29-0.38) | 0.03 |
| Impulsivity (W2) − Self-control (W2) | −21.96 (−24.63 to −19.30) | 2.05 | −0.49 (−0.53 to −0.44) | 0.02 |
| Impulsivity (W2) − Gaming time (W2) | 4.97 (0.94-8.99) | 1.36 | 0.08 (0.02-0.15) | 0.03 |
| Impulsivity (W2) − IGD (W2) | 3.61 (2.79-4.43) | 0.46 | 0.27 (0.22-0.33) | 0.03 |
| Self-control (W2) − Gaming time (W2) | −4.59 (−7.89 to −1.29) | 1.68 | −0.08 (−0.14 to −0.02) | 0.03 |
| Self-control (W2) − IGD (W2) | −3.76 (−4.47 to −3.06) | 0.36 | −0.30 (−0.35 to −0.25) | 0.03 |
| Gaming time (W2) − IGD (W2) | 3.65 (2.56-4.74) | 0.56 | 0.22 (0.16-0.28) | 0.03 |

Note. W1 = assessed at baseline, W2 = assessed at follow-up. The model was adjusted for school, age, sex, whether born in the study city, whether living with both parents, fathers’ and mothers’ educational level, and family financial situation.
